# Structural insight into the binding of C60-derivatives with enoyl-pyruvate transferase from Helicobacter pylori

**DOI:** 10.6026/97320630013185

**Published:** 2017-06-30

**Authors:** Mohammad Teimouri, Muhammad Junaid, Abbas Khan, Houjin Zhang

**Affiliations:** 1 Department of Biochemistry, Huazhong University of science and Technology, China; 2 Department of Bioinformatics and Biostatistics, Shanghai Jiao tong University, Shanghai, China

**Keywords:** C60-derivatives, Helicobacter pylori, enoyl pyruvate transferase, docking, prediction

## Abstract

Helicobacter pylori (H. pylori) is a human pathogen associated with acute gastritis and peptic ulcer. The MurA enzyme is an important drug target for the identification of ligands with improved efficacy and acceptable pharmaco-kinetic properties. We developed a homology model of H. Pylori MurA followed by refinement and molecular dynamics (MD) simulations. A total of 16 C60-derivatives were docked and its docking score were compared. Some of the known inhibitors were also similarly characterized and compared. Results show that five out of the sixteen C60-derivatives have good binding score. The MMPBSA analysis for the top five C60- derivatives shows good binding energy. This study reports the interaction patterns of selected C60 derivatives and MurA enzyme towards fullerene-based drug discovery.

## Background

A spiral shaped gram-negative bacteria; H. Pylori is responsible for different kind of diseases in Human population. In peptic ulcer and chronic active gastritis diseases H. Pylori is considered as the major causative agent.

A well-documented report regarding the association of H. Pylori with gastric inflammation, MALT, lymphoma and gastric adenocarcinoma are available [1]. Around 35% to 70% occurrence of gastric mucosa leading to gastric adeno-carcinoma has been reported. H. Pylori is also considered as the second leading cause of deaths around the world.

Cell wall of both gram negative and gram positive is precisely shaped with resistance to high osmotic pressure. Cell wall has its specificity for antibiotics such as penicillin and vancomycin [2]. Peptidoglycan biosynthesis machinery is considered as a target for developing new drugs against H. Pylori [3]. The mur family of enzymes catalyze several steps in the synthesis of peptidoglycan for bacterial cell wall [4]. MurA catalyzes the first step while MurB catalyzes the reduction to D-lactate. The reduction produces UDP-N-acetylmuramate. MurC, MurD, MurE and MurF are the other enzyme that helps in the completion of this pathway. The present study targets the MurA enzyme. The synthesis of structural element of murein is initiated by the unusual transfer of enol-pyruvyl from phospho-enol-pyruvate (pyruvate-P) to 3-hydroxyl of UDP-N- acetylglucosamine (UDPGlcNAc) catalyzed by UDP-GlcNAc enol-pyruvyl-transferase (MurA).

A large number of antibiotics are available for the inhibition of this pathway. However, few inhibitors are available for the first step. The x-ray crystallographic structure of MurA is not known. Therefore, it is of interest to develop the 3D model of the MurA enzyme using the MurA of Pseudomonas aeruginosa having 47% identity as template. The water-soluble C60 (also known as fullerene) derivatives were further docked into the active of MurA model for the identification of improved inhibitors. The C60 derivatives show good binding affinity towards the active site. We describe the binding mode of C60 derivatives with MurA in this study.

## Methodology

### Template selection

Universal Protein Resources (Uniprot) was used to retrieve the primary sequence of MurA using the accession number Q9ZLI6 (http://www.uniprot.org/). PSI-BLAST was further used to select the best templates for the query sequence [5]. MUSCLE was then used for the alignment between the query sequence and selected template.

### Model generation of Enoyl-pyruvate transferase (MurA)

Online servers were used to generate the 3D structure but the final model was built using MOE 2014. Around 100 models were generated and were optimized. Amber99 force field along with the GB/VI scoring function [6] was used for energy minimization. Subsequently, a short molecular dynamics simulation followed by energy minimization in order to refine the structure was completed.

### Preparation of fullerene derivatives

**Figure 1:**
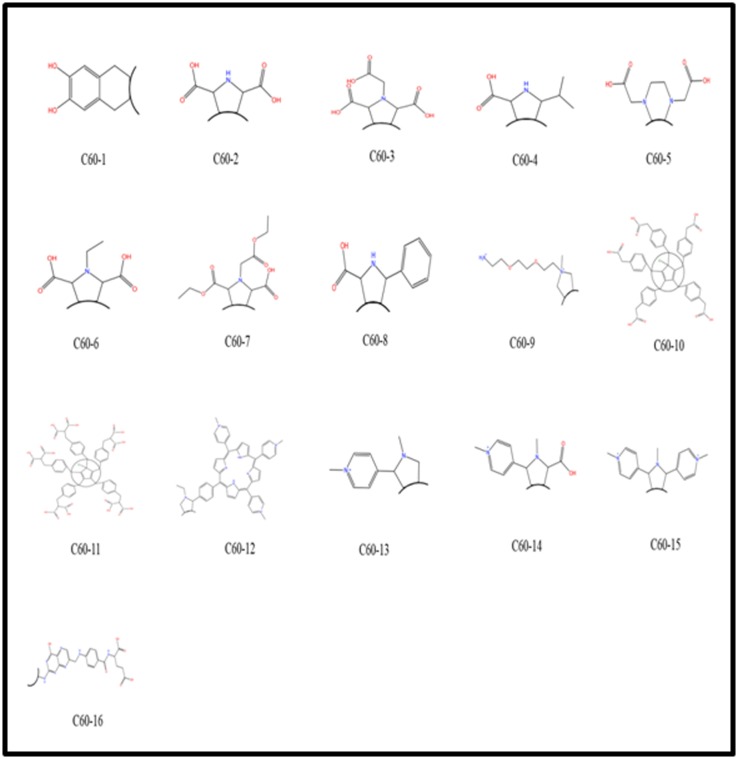
Two-dimensional structure of water-soluble C60 derivatives. The Arc in each structure shows the attachment of fullerene ball.

The literature was searched to collect all known fullerene derivatives reported by different authors (Figure 1) [7-14]. The three dimensional structure of ligands were drawn by using the Builder software implemented in MOE2014 and a ligand database was constructed. Partial charges were calculated for all the molecules using the Merck Molecular Force Field 94X (MMF94X) which is suitable for small molecules [15]. Subsequently, the energy of all fullerene derivatives was minimized with a convergence criterion = 0.05 kcal/mol Å2 using the conjugated gradient Newton optimization algorithm.

### Molecular Docking

The docking of C60 derivatives against the MurA enzyme was carried out using MOE. The modeled structure of the MurA was used as input for the docking purpose. The correct protonation state was assigned to each residue by using the protonated 3D module embedded in MOE. The whole structure of each enzyme was used as a receptor to find the potential binding sites. London dG scoring function was used to calculate free energy of binding of given conformation of the ligand in the active site.

### Binding Energy and Binding Affinity Calculations

Binding affinities were calculated to identify the best ligand. Generalized Born/volume integral (GB/VI) implicit solvent method was used. The estimated binding affinity was computed using the GBVI/WSA dG scoring function calculated in Kcal/Mol units. Energy minimization of binding pocket in each fullerene derivative complex was performed before calculating binding affinity.

### Molecular Dynamics (MD) Simulation

The MD simulation was carried out for each complex using amber14 software with ff14SB force field. The system was made neutral by adding the Na+ counter ions. The complex structure was solvated with TIP3P water molecules in a rectangular box with 8.0 Å buffer distances for each side. Subsequently, the energy of the system was minimized in two steps using sander module of amber14. First, the energy of the water and counter ions was minimized keeping the protein fixed using harmonic constraint of strength of 500 kcal mol-1Å-1. Secondly, all atoms were energy minimized without restriction. In each step, the steepest descent minimization was followed by the conjugate gradient minimization. The steps consist of 2000 and 4000 respectively. Then, the system was heated from 0 to 300 K in 500 ps and equilibrated at 300 K for another 500 ps. Finally; the system was simulated for the 50 ns. The simulation was conducted at constant pressure and 300 K. The coordinates were saved every 10,000 steps.

## Results and Discussion

### Template selection and model building

**Figure 2:**
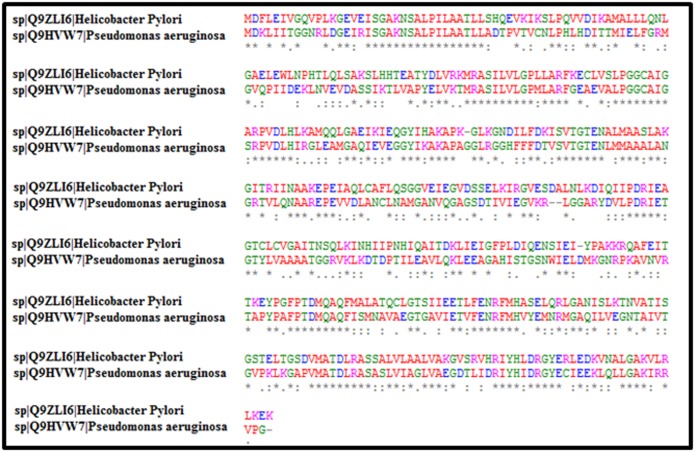
Sequence alignment of enoyl pyruvate transferase of H. pylori and Pseudomonas aeruginosa. The sequence alignment shows that both the sequences share 47% identity.

**Figure 3:**
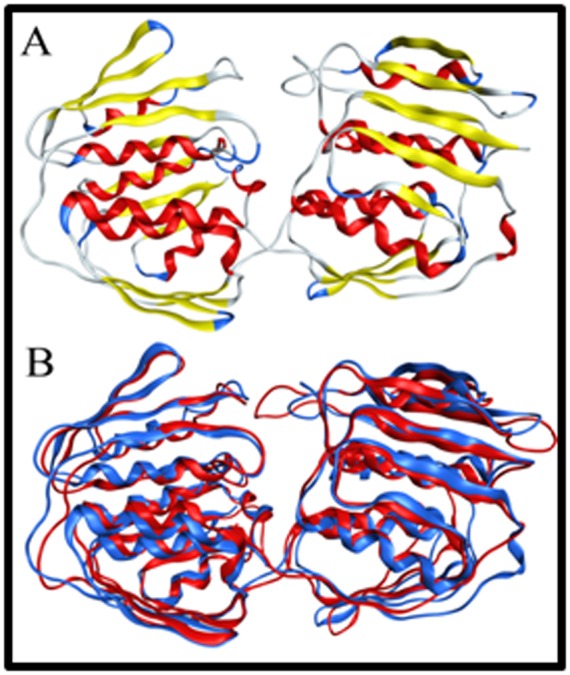
(A) A homology model of enoyl pyruvate transferase (B) Superposition of template (Blue) and homology model (Red) is shown. The superposition was completed using the MOE software. Superposition shows close similarity between the template and the target protein.

Among the homologous proteins selected by BLAST, UDP-Nacetyl- glucosamine 1-carboxy-vinyl-transferase of Pseudomonas aeruginosa (PDB ID 5BQ2) was used as template having 47% similarity with the target protein for model generation. The alignment of 5BQ2 and H. pylori Enoyl pyruvate transferase is given in Figure 2. Hundred intermediate models were superimposed and negligent differences were noticed in secondary structure among them. There is a difference in contact energies, GB/VI scoring and potential energy so the model with the best MOE packing score (contact energy -377.6724, Packing score 10.7361, GB/VI -17621.337 and potential energy -6237.1777) is selected for further analysis. The generated model contain two globular domain connected by a loop and each composed of eight alpha helix surrounded by twelve beta sheets (Figure 3A). The modeled structure was superimposed on to the template, giving root mean square deviation (RMSD) of 1.8 angstrom (Figure 3B), showing close structural similarity with template protein.

### Molecular Docking and binding affinity calculation

**Figure 4:**
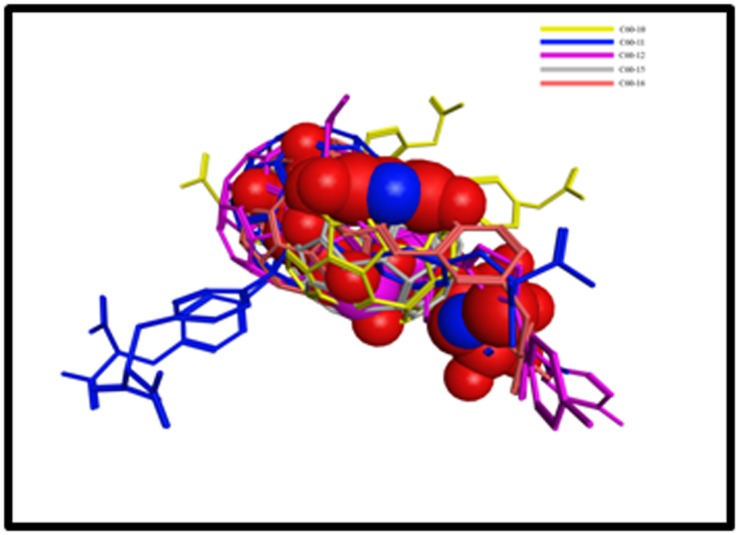
Superposition of the top C60-derivatives. The top C60-derivatives bound to the active site and they superpose well on the reported inhibitor (compound 1). The best reported inhibitor is shown in sphere model while C60 derivatives is shown in ball and stick model.

To explore the binding mode of each compound, all compounds were docked into the active site of the enzyme. Several classes of inhibitors of MurA were reviewed in the literature [16]. All reported inhibitors of MurA were collected from literature and were docked into the active site of MurA. The docking score and binding affinity of all the reported inhibitors was calculated (Table 1) and were used as standard to screen the best C60 derivatives. Out of sixteen C60 derivatives, only five derivatives were found to have good binding affinity and docking score. The active site of MurA is formed by the groove and can accommodate the large molecule. The better binding affinity and docking score of the compound-1 may due to its large interface area with active site. The five C60 derivatives superimpose well on each other and bind the same binding site as occupied by the substrate (Figure 4). So, these derivatives may block the access of the substrate to the active site and hence reduce or may inhibit the activity of enzyme.

Docking data show that C60-10 was found to have better binding affinity and docking score than previously reported inhibitors and other C60-derivatives. The binding affinity and docking score of C60-10 is 23.059 pKi and -33.87, respectively. C60-10 makes several hydrogen bonds with the residues lining the active site. The residues include L22, N23, R93, E169, D235, R236 and G401. The binding mode of the C60-10 blocks the access of substrate to the active site. Several hydrophobic interactions were also observed consisting of L26, L97, I119, V165, I193, I237, L330, F331 and L373 (Table 2). Among the reported inhibitors, the compound-1 has good docking score and binding affinity -18.67 and 11.954 pKi. The binding mode of compound-1 shows that it make hydrogen bonds with residues L22, R93, A94, A121 and R122. In comparison to compound-1, the C60-10 has a better hydrogen-bonding network that may contribute to better binding affinity and docking score.

From the results of molecular docking, the C60-11 was found to have good docking score and binding affinity after C60-10. The C60-11 bound tightly with binding affinity and docking score 22.619 pKi and -32.62 respectively. The C60-11 makes strong hydrogen bonding network with the active site. The residues making hydrogen bonding network includes I163, D235, R236, T307, E332, N333 and T371. The side group of C60-10 and C60-11 has several carboxylic groups attached (Table 2) that may contribute for the strong hydrogen-bonding network. The side groups attached to the C60-15 and C60-16 are small and were found to have comparatively low docking score and binding affinity (Table 2). The docking results showed that comparatively large side groups on the C60 molecule favor strong binding in the active site.

### Molecular dynamics simulation

**Figure 5:**
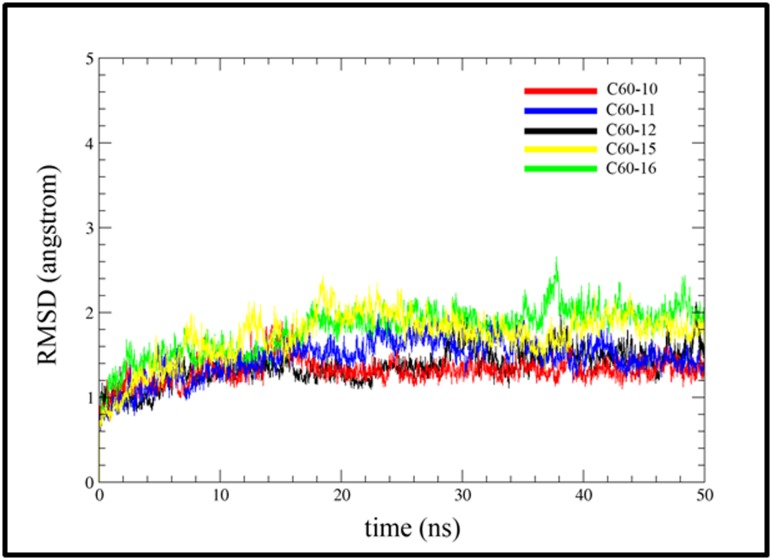
RMSD versus time graph of alpha carbon during the 50ns of simulation. The RMSD converged around 15ns for all the C60 derivatives and remained stable there after.

In this section, the structural analysis was completed over 50 ns trajectories for each system. The stability of each system was checked using the root mean square deviation (RMSD). Figure 5 shows the root mean square deviation of each system for Cα atoms. As shown by Figure 5, the deviations of RMSD for all the system are within the 2 Å. This shows that the MurA enzyme suffer no significant changes in the structure. Each C60 - derivative was found stable in the active site of MurA. The RMSD values increased in the first 20 ns and then fluctuate within a narrow range for the rest of simulation. The plot in Figure 5 shows that the system reached equilibrium after 20ns. As expected, the lowest RMSD was shown by the C60-10 complex. This may be due to the larger size of C60-10 that fit well into the active site of MurA and enable it to have numerous interactions with the active site (Figure 5). A large number of hydrogen bonds and hydrophobic interactions make the C60-10 stable in the active site. The second lowest RMSD was shown by the C60-11. The C60-11 has an extra carboxyl group at each side (Table 2). Due to these extra bulky groups, the RMSD of the C60-11 is more than C60-10 to adjust itself in the active site. The last 20 ns simulation shows that C60-11 is stable as C60-10. The RMSD plot of C60-15 and C60-16 is comparatively high.

### MMPBSA analysis

The structural analysis showed that C60 fullerene derivatives block the active site of MurA. This was supported with the MMPBSA approach. The goal of MMPBSA calculation was to find out the binding free energy of C60 fullerene derivatives with the active site of MurA. Results show that the van der waals interactions were mostly contributed to binding energy in C60 derivatives. The polar solvation energy, which is an unfavorable contribution to the binding free energy, appeared to be highly positive. The order of the ΔGbinding was found to be same as pKi calculation. The order is C60-11(-81.8915) > C60-10(-71.3011) > C60-12(-49.7352) > C60-15(-37.4502) > C60-16(-37.4966). The van der Waals, electrostatic interactions and non-polar solvation energy contribute negatively to the total ΔG binding, while only the polar solvation energy is unfavorable, with positive value (Table 3).

## Conclusion

A homology model of the MurA enzyme from H. Pylori was developed, refined and simulated over 50ns for structure geometry validation. The active site residues were subsequently mapped on to the structure model for further docking study. The residues L22, R120, R331 and R371 of E. coli corresponding to L22, R122, R334, and R374 of H. Pylori were mapped. Subsequently, results from the docking of C60 derivatives with the MurA enzyme model were reported in this study. The binding affinity and docking score of compound #10 was 23.059 pKi and -33.87, respectively. Few other C60 derivatives also showed good binding affinity. Compound #10 was followed by #11 with a docking score of -32.62 and binding affinity of 22.619. Compounds #15 and #16 also showed acceptable score. These data finds application in the design of a suitable inhibitor against MurA. It should be noted that these prediction data should be further evaluated using toxicity studies followed by in vitro and in vivo models and their analysis.

## Figures and Tables

**Table 1:** analysis of the reported inhibitors against MurA

Compound ID	Docking score	Binding affinity (pKi)	Interacting residues	
			Hydrogen Bond	Hydrophobic
Compound-1	-18.67	11.954	K22, R93, A94, A121, R122	I119, V165, I237, L330, F331,
Compound-2	-10.11	9.452	N23, D235,	V165, I237
Compound-3	-9.53	7.421	R122	I163, V165, L330
Compound-4	-9.13	7.493	R122, T329,	I163, V165, I237, L330
Compound-5	-9.08	7.706	R122, E301, T329	I163, V165, L330
Compound-6	-8.51	6.637	A121, T166,	L330
Compound-7	-7.87	6.079	T166	L330
Compound-8	-7.58	6.422	I163, V165, L330	-
Compound-9	-7.14	5.65	T166	V165, L330, F331
Compound-10	-7.06	4.695	S164, V165, T166	V165, L330
Compound-11	-6.98	5.157	I163, V165, L330	-
Compound-12	-6.34	5.714	R122, T329,	I163, V165

**Table 2:** Docking analysis of the top C60 derivatives against MurA

Compound Name	Docking score	Binding affinity (pKi)	Interacting residues	
			Hydrogen Bonded	Hydrophobic
C60-10	-33.87	23.059	K22, N23, R93, E169, D235, R236, G401,	L26, L97, I119, V165, I193, I237, L330, F331, L373
C60-11	-32.62	22.619	I163, D235, R236, T307, E332, N333, T371	L26, I119, V165, L330, F331, L373
C60-12	-32.45	18.064	T166	L26, I119, I163, V165, I237, L330, F331, L373, E190, D308
C60-15	-22.56	12.704	N23	L26, I119, V165, L330, F331, L373, D49, E192, D235
C60-16	-25.01	12.659	T166, E192, T307, D308	L26, I119, V165, L330, F331, L373

**Table 3:** The MMPBSA analysis of the final hit compounds of C60-derivatives

Compound	MM/PBSA (kcal/mol)				
	Electrostatic energy	van der Waals energy	Non- polar energy	Polar energy	ΔGBIND
C60-11	-53.0279	-106.9794	-7.2696	95.9859	-71.3011
C60-12	-304.555	-141.7395	-9.7581	373.6085	-81.8915
C60-13	-36.9207	-113.1473	-9.7601	109.4807	-49.7352
C60-16	-82.3407	-62.674	-5.4182	113.1447	-37.4502
C60-17	-94.2365	-66.0277	-5.2953	128.2149	-37.4966
